# Emotional health, support, and self‐efficacy in young adults with a history of language impairment

**DOI:** 10.1111/bjdp.12148

**Published:** 2016-05-25

**Authors:** Nicola Botting, Kevin Durkin, Umar Toseeb, Andrew Pickles, Gina Conti‐Ramsden

**Affiliations:** ^1^ City University LondonUK; ^2^ University of StrathclydeGlasgowUK; ^3^ The University of ManchesterUK; ^4^ Kings College LondonUK

**Keywords:** developmental disorder, language impairment, depression, anxiety, support, self‐efficacy

## Abstract

Children and adolescents with language impairment (LI) are at risk of emotional health difficulties. However, less is known about whether these difficulties continue into adulthood for this group, or about the potential role of environmental resources (e.g., social support) or internal resources (e.g., self‐efficacy). This study investigates emotional health in 81 adults with a history of developmental LI (aged 24) compared with 87 age‐matched peers (AMPs) using Beck Inventories. Social support and self‐efficacy measures were examined as predictors. The results were fourfold: (1) adults with LI had higher levels of emotional health problems; (2) whilst the availability of social support was similar across groups, people with LI received more help from others compared to peers; (3) social support was not significantly related to emotional health in those with LI – in contrast, for AMPs, uptake of support indicated poorer emotional health; (4) self‐efficacy was the strongest predictor of emotional health in both groups and fully mediated the relationship between language and emotional health (no moderation by group). This cross‐sectional study has implications for concurrent factors that might affect emotional health outcomes for children and young people with and without LI.

## Background

Individuals with developmental disorders may be particularly at risk of emotional health difficulties. Children and adolescents with language impairment (LI) experience higher levels of depression and anxiety than in typical populations (Conti‐Ramsden & Botting, 2008) and this developmental context suggests that similar difficulties are likely to persist into adulthood. Indeed, higher levels of depression and anxiety have been reported in other adult groups with developmental disorders (compared to typical peers), such as those with autism (Lugnegård, Hallerbäck, & Gillberg, 2011) and those with attention deficit hyperactivity disorder (ADHD; Nelson & Gregg, 2012). Relevant research with people with LI, however, is scant. Identifying correlates and predictors of emotional health in adults with LI entails addressing whether environmental resources, such as social support, and/or internal resources, such as self‐efficacy, have significant influences on the way in which comorbid emotional health problems present. Emotional health difficulties are expensive to treat once they reach clinical levels (Thapar, Collishaw, Pine, & Thapar, 2012). A better understanding of the associations between social support, self‐efficacy, and emotional health among those with a history of developmental LI will contribute to our broader understanding of mental health in people with developmental disorders and may facilitate more effective targeting of preventative and/or protective strategies. In this study, we investigate these variables in a sample of young adults with histories of developmental LI and compare them to a sample of age‐matched peers (AMPs) without LI.

### Emotional health and language difficulties

The connection between language and emotional health at young ages is well established (see Durkin & Conti‐Ramsden, 2010; Toppelberg & Shapiro, 2000 for overviews). Higher levels of emotional health difficulties have been reported in children with LI (Cantwell & Baker, 1987; Maggio *et al*., 2014) and adolescents with LI (Beitchman *et al*., 2001; Conti‐Ramsden & Botting, 2008; Snowling, Bishop, Stothard, Chipchase, & Kaplan, 2006; Voci, Beitchman, Brownlie, & Wilson, 2006; Wadman, Botting, Durkin, & Conti‐Ramsden, 2011).

The precise mechanisms for the link between emotional health and language abilities are not entirely clear. Possible developmental factors that are likely to be involved fall into two main categories: gene–environment influences and internal child factors. Gene–environment influences may include the fact that parents of children with LI experience higher rates of emotional health problems which in turn may impact on the level of family support available to children and young people (O'Connor, Heron, Golding, Beveridge, & Glover, 2002). Furthermore, developing peer relations can also be problematic for children with LI (Leve, Kim, & Pears, 2005; Mok, Pickles, Durkin, & Conti‐Ramsden, 2014) and friendships may be more difficult to form (Durkin & Conti‐Ramsden, 2007). There is, however, not enough evidence to disentangle and specify the extent of the contribution of environmental support and/or genetic predisposition to mechanisms responsible for the association between emotional health and LI (see also Conti‐Ramsden & Botting, 2008 for a discussion of these issues). Internal child factors are also implicated, for example research with children with LI suggests that language difficulties may impact on how children comprehend emotional descriptions and how well they can self‐regulate their emotions (Beck, Kumschick, Eid, & Klann‐Delius, 2012; Fujiki, Brinton, & Clarke, 2002; Spackman, Fujiki, & Brinton, 2006). Having a language difficulty may also affect resilience to emotional health difficulties, both at a personality level (e.g., self‐efficacy) and at a neurological one (e.g., a comorbid deficit).

There is limited research addressing emotional health in adults who have grown up with developmental LI (Beitchman, Brownlie, & Bao, 2014; Clegg, Hollis, Mawhood, & Rutter, 2005; Records, Tomblin, & Freese, 1992; Whitehouse, Watt, Line, & Bishop, 2009). What is available has yielded mixed evidence about whether risk continues beyond teenage years. Clegg *et al*. (2005) and Whitehouse *et al*. (2009) all found that emotional health problems were manifest, but Beitchman *et al*. reported that diagnoses of affective disorder in a sample of adults in their early 30s were not significantly more prevalent than in a comparison group without LI. Records *et al*. (1992) similarly found no difference on quality of life measures between adults with and without LI. The question thus remains as to whether adults who have grown up with developmental LI are more susceptible to emotional health difficulties and what are the likely factors involved at this stage of their lives.

### Social support and self‐efficacy in depression and anxiety

Social support is likely to be an important consideration when documenting risk in emotional health. In the literature on typical adults (Aneshensel & Stone, 1982), as well as in older adults with acquired language difficulties (Hilari, Needle, & Harrison, 2012), perceived social support provides an important context for ameliorating depression and anxiety. Despite these findings, measurement of social support is not straightforward. Perceived social support centres on an individual's own ratings and sense of available support. This may be different from the actual amount of support received when objectively quantified, for example, in terms of time or instances when reported by a significant other. There may also be important qualitative differences in who provides support for healthy adults and for those from clinical groups. Although to our knowledge, no previous studies of emotional health in adults with LI have included social support measures, findings from adolescents have suggested that family may play a larger role for these individuals compared to peers (Botting & Conti‐Ramsden, 2008; Conti‐Ramsden & Durkin, 2008).

Explanations of associations between social support and emotional health are likely to be multifactorial: On the one hand, those with poorer emotional health may seek support more often; on the other hand, individuals with higher social support may experience fewer symptoms. Thus, it is important to explore whether different patterns of association occur within groups with LI. The cause of the emotional health issues are not clear in young people with LI. Conti‐Ramsden and Botting (2008) have previously argued that in young people with LI, emotional health might be part of a neurodevelopmental trajectory rather than resulting from poor communicative experiences *per se*, whereas ‘loss of skills’ is sometimes cited by clinicians and service users as a cause of sadness and worry in those with acquired LI. This population difference may affect the experience of emotional health in adulthood and is likely to be relevant to the relationship between social support and depression/anxiety.

At the same time, specialized support for young adults with LI once they become independent is rarely available. Speech‐language services in the United Kingdom provide support only up to the age of 19 (although in the US support continues until 21 years of age and recent UK changes mean that individual plans may extend until 25 years of age). However in practice, many individuals across different countries lose direct specialized support long before adulthood. Although there are learning disability and neurorehabilitation services for adults, these provisions address the needs of individuals with global delay or aphasia‐/dementia‐related language difficulties. Despite this lack of resources for young people with LI, older adolescents have reported strong supportive roles for community‐based initiatives such as specialist youth groups. Myers, Davies‐Jones, Chiat, Joffe, and Botting (2011), for example, reported that young people up to the age of 20 felt supported by attendance at a group designed specifically for those with developmental communication problems. Very few such resources exist, however, and people with LI often feel adrift as they get older and attempt to achieve independence and enter the world of work (Joffe, Beverly, & Scott, 2011). In this context, social support may become even more salient.

As well as social support, internal feelings of control and self‐efficacy come to the fore as important factors. Self‐efficacy is the conviction that one can achieve personal goals independently. The concept originates from Bandura's Social Learning Theory (1986) where self‐efficacy is placed as an important factor in learning (Bandura, 1997) and career trajectories (Bandura, Barbaranelli, Caprara, & Pastorelli, 1996, 2001). Self‐efficacy ratings have been found to associate with academic achievement (Bassi, Steca, Delle Fave, & Caprara, 2007), shyness (Caprara, Steca, Cervone, & Artistico, 2003), career development, and emotional health in the general population (Lucas, Skokowski, & Ancis, 2000). Importantly, higher levels of self‐efficacy seem to act as a protective factor for depression in children and adolescents (Smith & Betz, 2002; Steca *et al*., 2014). It is plausible that having a developmental LI is associated with increased experiences of ineffectualness in adulthood. Everyday tasks are noticeably more difficult in the context of poor language, and this may result in low perceived self‐efficacy, which in turn may result in poor emotional health. However, to our knowledge, self‐efficacy has not been explored in young adults with LI. Thus, it is not clear whether self‐efficacy is related to emotional health in the same way for adults with LI compared with AMPs.

### The present study

The present study examines the levels of depression and anxiety in a large group of young people aged 24 years who have grown up with developmental LI compared with AMPs without a history of LI. We sought to clarify mixed results from other studies as to whether adult risk of emotional health exists in this group (Beitchman *et al*., 2014; Clegg *et al*., 2005; Whitehouse *et al*., 2009) and to investigate for the first time the relationships between social support, self‐efficacy, and emotional health. Because the issues around social support are complex and because they are likely to be different at different stages of development, the present study uses a cross‐sectional design to shed light on outcomes of development, namely LIs. This is an important first step that can inform research on potential longitudinal effects in adulthood of growing up with language difficulties.

Specifically, our research questions are as follows:

1. Are levels of depression and anxiety higher in young adults with a history of LI compared to AMPs?
2. Do concurrent environmental factors, such as the availability or receipt of support, relate to depression and anxiety?
3. Do concurrent internal factors, such as self‐efficacy, act as a protective factor against depression and anxiety? Is this different for those with LI compared to AMPs?

## Method

### Participants

Two groups of young adult participants (aged 24) were recruited from within the large‐scale longitudinal research programme referred to as the Manchester Language Study (Conti‐Ramsden & Botting, 1999a; Conti‐Ramsden, Crutchley, & Botting, 1997): those with a history of developmental LI and AMPs. The groups were compared cross‐sectionally to assess any differences and to examine relationships between concurrent variables.

#### Young people with LI

The initial cohort of 242 children with LI was originally recruited at 7 years of age as having primary language difficulties. There were originally 186 boys (77%) and 56 girls (23%) in the sample, representing a random 50% sample of all 7‐year‐olds attending specialist language classes in England. At recruitment, 53% could be classified as having expressive–receptive difficulties, 38% expressive only difficulties, and 9% primary pragmatic language difficulties (Conti‐Ramsden & Botting, 1999a). Although the current study investigates the outcomes of these children in adulthood using a cross‐sectional design, it is important to note that the sample was recruited in childhood and remains representative of the group of young people with a history of developmental LI: There were no significant differences in receptive or expressive language nor performance IQ (PIQ) at age 7 between those who participated at age 24 and those who did not (all *p*‐values > .2). Recruiting from a longitudinal sample is important even when considering outcomes cross‐sectionally, because we know that some language and cognitive change occurs in this group (see Botting, 2005; Conti‐Ramsden & Botting, 1999b), and therefore, assessment of outcome in adulthood leads to a selective sample of individuals with the most persistent profiles. In total, 81 participants (54 males, 27 females) with a history of LI were included in the analyses presented here, representing those who had complete depression and anxiety data at 24 years of age. Attrition was higher for males compared with females, χ^2^(1) = 7.5, *p* = .006, but the distribution of males:females was not significantly different from the AMP group (Fisher's exact *p* = .16).

#### Age‐matched peers

The comparison group comprised 87 AMPs (48 males, 39 females) with data for both depression and anxiety at 24 years of age. These participants had no history of special educational needs or speech and language therapy provision. Groups did not differ on age, gender, household income at age 16 when the AMP group was recruited (*p* = .80) nor personal income at age 24 (*p* = .40). As expected, language and PIQ profiles were different across the groups (see Table 1).

**Table 1:** Psycholinguistic characteristics of participants

	Age	Gender (% male)	CELF core language index	WASI non‐verbal IQ
LI	24; 4	66.7	69.9 (20.5)	98.8 (16.1)
AMP	24; 0	55.2	100.0 (13.9)	111.9 (10.3)

Note

AMP = age‐matched peer; CELF = Clinical Evaluation of Language Fundamentals; LI = language impairment; WASI = Wechsler Abbreviated Scale of Intelligence.

Values are means and *SD* unless otherwise stated.

### Measures

#### Language

The Clinical Evaluation of Language Fundamentals (CELF‐4^uk^; Semel, Wiig, & Secord, 2006) was used to assess language ability. Given the dearth of standardized language tests in adulthood, the CELF‐4 was deemed the best fit assessment for our cohort at 24 years of age since this assessment is normed up to 21; 11 (and in fact neither group reached ceiling levels on this assessment). A core language index was created using standard scores (based on 21; 11 year norms) from the Recalling Sentences, Formulated Sentences, and Word Classes subscales.

#### Non‐verbal IQ

The Wechsler Abbreviated Scale of Intelligence (WASI, Wechsler, 1999) Performance subscale was administered as a measure of non‐verbal IQ and standard scores were calculated. This test has norms for individuals aged 6–89 years. The reliability of the PIQ scale for the age range 20–24 years is .94.

#### Emotional health

Emotional health was measured using Beck Depression Inventory II (BDI; Beck, Steer, & Brown, 1996) and the Beck Anxiety Inventory (BAI; Beck & Steer, 1993) as the primary outcome measures. The BDI questionnaire consists of 21 items across depression symptoms including: sadness, pessimism, past failure, loss of pleasure, guilty feelings, and suicidal thoughts. For each item, there are four statements differing in severity and coded 0 for no symptoms to 3 for severe symptoms. Participants were asked to choose the statement that best describes them during the past 2 weeks. For the BAI, 21 items were presented to participants, each consisting of one statement (e.g., fear of losing control), for which the participant was asked to rate experience of that symptom for the past week. A 4‐point scale was used, where 1 was ‘not at all’, and 4 was ‘severely – I could barely stand it’. Participants were presented with the response options visually, and items were read out loud. The reported internal reliability of the BDI is α = .81 (Beck, Steer, & Carbin, 1988b) and of the BAI is α = .92 (Beck, Epstein, Brown, & Steer, 1988a).

#### Support and community integration

Several different measures of support were obtained both from respondents and from a significant other nominated by the participant (LI *n* = 80; AMP *n* = 86). In the majority of cases, this person was a parent (LI *n* = 71; AMP *n* = 66), but in a few cases was a sibling (LI *n* = 3; AMP *n* = 8), partner (LI *n* = 5; AMP *n* = 7), or friend (LI *n* = 1; AMP *n* = 5).

##### Self‐reported social support

A number of measures of social support were obtained. The first measure was an adapted version of the Personal Resource Questionnaire part 1 (PRQ85; Brandt & Weinert, 1981). This adapted scale, which consisted of 11 items, asked about support across a range of problem situations: crisis, partner, family, friend, financial, loneliness, illness, upset about life condition, work, dealing with official documentation, and general administration. For each scenario, participants were asked: (1) which types of *available support* would be available for that scenario – participants were able to choose from a list of possible support streams which were as follows: parent, partner, other family member, friend, neighbour or colleague, spiritual advisor (e.g., minister), professional (e.g., counsellor), agency (e.g., citizen's advice bureau), Internet support – (2) whether that problem had occurred in the past 6 months. For all of these data points, a sum was made for each participant for (1) above: the total *available support* across all problems. This was done by summing the number of possible sources of support indicated for each problem; and for (2) above total *problems in past 6 months*, a variable created by totalling the number of different types of issues in the PRQ85 that had occurred within that time. These summed scores were used as the key self‐reported social support outcome variables.

Participants also rated how often they accessed more formal, *organized support* systems on a 5‐point scale from ‘never’ (0) to ‘most days’ (5). Participants were asked about support from: library, citizens' advice, health visitor/GP, union, community centre, debt‐help organizations, Samaritans, alcohol/drug charities, homeless charities, health support groups, carer support groups, social workers, place of religious worship, and other. The scale was used to measure the frequency of support from a variety of different sources.

Community integration was assessed using the Community Integration Measure (McColl, Davies, Carlson, Johnston, & Minnes, 2001). The scale consists of 10 statements for example, ‘There are always people I feel close to in this community’. Participants rated these statements on a 5‐point scale from ‘always agree’ (5) to ‘always disagree’ (1). The scale had good internal reliability in our sample (α = .8).

##### Nominated person reported social support

The nominated person was asked about support in two ways. Firstly, they were asked to rate how much help/support he or she believed the participant received from others including themselves using a 7‐point scale where 1 represented ‘never gets help/support’ to 7, which represented ‘Always gets help/support’. This scale is referred to as the *other‐perceived support score*. This nominated person also stated whether she or he personally helped the participant regularly (yes/no) in respect of five different scenarios. These scenarios were as follows: practical errands, social situations, finance or money, reading or writing, and emotional issues. These were summed to give a *support received from nominee* score.

Overall the support measures totalled six key scales: four that were self‐report (available support from PRQ85; problems in past 6 months from PRQ85; organized support; and community integration) and two that were completed by nominated persons (other‐perceived support; support received from nominee).

#### Self‐efficacy

The General Self‐Efficacy Scale (Schwarzer & Jerusalem, 1995) was used. This is a scale consisting of 10 statement items relating to self‐efficacy (e.g., I can always manage to solve difficult problems if I try hard enough). Participants rated each statement on a 4‐point scale where 1 was ‘not at all true’ and 4 was ‘exactly true’. The scale had good internal reliability in our sample (α = .9).

### Procedure

The study was granted ethical approval by the University of Manchester Research Ethics Committee. All participants gave written informed consent to take part in the study and also consent to contact the nominated respondent. Written consent from the nominated participant was also gained. All measures were completed as part of a face‐to‐face interview, which took place in the participant's home or at an arranged location. Wherever possible, the participant was alone to ensure confidentiality. The researcher delayed emotional health questions until there was sufficient privacy or asked the participant whether they would prefer to answer them without being overheard. All items were read out loud to the participants who were also provided with a visual display of the possible responses.

### Statistical analysis

All statistical analyses were conducted in Stata/SE 13.1 (StataCorp, 2013). A two‐tailed significance level of *p* = .05 was used unless otherwise specified. Independent samples *t*‐tests were used to compare group differences in measures of emotional health, social support, and self‐efficacy. Pairwise correlations were run to test zero‐order associations between the variables of interest. This was done separately for the LI group and the AMP group. Next, the stepwise method for regression analyses was conducted to establish predictors of depression and of anxiety. For both models, seven predictors (six social support variables and self‐efficacy) were entered in the first step. Non‐significant predictors (*p* > .05) were removed from the model and the models were re‐run with a dummy variable for group also as a predictor. It is important to note that the term ‘predictors’ used here refers to concurrent statistical predictors rather than developmental ones which would require examination of longitudinal data across different time points. Self‐efficacy was investigated further using a more specific mediation analysis following Baron and Kenny (1986). The mediating effect of self‐efficacy on the relationship between language ability and emotional health (composite of BAI and BDI) was investigated. Then, group (LI or AMP) was entered as a moderator in the relationship between language and self‐efficacy.

## Results

Group differences were seen in areas of emotional health, social support, and self‐efficacy (Table 2).

**Table 2:** Group differences in emotional health, social support, and self‐efficacy

	Mean (*SD*)		*t*‐test	Mean diff (95% CI)	Effect size, *d*
	LI	AMP			
Depression	9.8 (9.1)	6.4 (7.2)	*t*(152.8) = 2.7**	3.4 (0.9, 5.9)	−.4
Anxiety	7.8 (7.5)	5.3 (8.3)	*t*(166) = 2.0*	2.5 (0.1, 4.9)	−.3
Available support	22.7 (10.6)	23.0 (10.9)	*t*(165) = −0.1	−0.2 (−3.5, 3.1)	.0
Number of problems last 6 months	2.4 (2.2)	2.2 (2.3)	*t*(165) = 0.4	0.1 (−0.6, 0.8)	−.1
Organized support	13.54 (1.1)	13.6 (0.9)	*t*(165) = −0.2	−0.0 (−0.3, 0.3)	.0
Community integration	39.6 (7.1)	42.1 (6.5)	*t*(165) = −2.4*	−2.6 (−4.6, −0.5)	.4
Other‐perceived support	3.2 (1.9)	2.3 (1.6)	*t*(164) = 3.3**	0.9 (0.4, 1.4)	−.5
Total amount of support received from nominee	2.2 (1.5)	1.2 (1.1)	*t*(140) = 4.9***	1.0 (0.6, 14)	−.8
Self‐efficacy	29.4 (5.6)	32.7 (4.1)	*t*(143.7) = −4.4***	−3.3 (−4.9, −1.8)	.7

Note

AMP = age‐matched peer; LI = language impairment.

Depression, total amount of support received from nominee, and self‐efficacy corrected for unequal variances.

*p < .05; **p < .01; ***p < .001.

### Group comparisons of emotional health

The mean depression and anxiety scores for young adults with LI were higher than for the AMP group. There were significantly more people in the LI group with a clinical level of depression (score > 19 on BDI: 14.8%) compared with AMP (3.4%; Fisher's exact *p* = .013). For anxiety, there were slightly more individuals with LI scoring over the clinical cut‐off of 15 on the BAI but this fell short of significance, LI: 18.5%; AMP: 8.0%, χ^2^(1, *N* = 168) = 4.04, *p* = .066. In the LI group, 9/81 individuals were above cut‐offs for both depression and anxiety compared with 3/87 of the AMP group.

### Group comparisons of support and self‐efficacy

There were no significant differences between groups in the amount of *available support*, the *number of problems in past 6 months*, or the use of *organized support*. Moreover, when these data were examined descriptively, the nature of the support networks was similar for each group: Participants in the LI group were most likely to choose parents as a support for 8/11 scenarios, and for the AMP group, this was true for 6/11 scenarios. For LI and AMP, friends, partners, and relatives were the next most common groups to whom they would turn to for help.

Young adults with LI were, however, significantly less integrated into their communities and received more support as rated by the nominated person on the *other‐perceived support scale*, as well as in terms of *support received from nominee*. The young people with LI reported less perceived self‐efficacy than the AMP group.

### Associations between emotional health, support, and self‐efficacy

The associations between emotional health, social support, and self‐efficacy are presented in Table 3.

**Table 3:** Zero‐order associations between variables

	1.	2.	3.	4.	5.	6.	7.	8.	9.	10.	11.
1. Depression	1										
2. Anxiety	LI: .7*** AMP: .7***	1									
3. Emotional health composite	LI: .9*** AMP: .9***	LI: .9*** AMP: .9***	1								
4. Available support	LI: −.1^NS^ AMP: .2^NS^	LI: .0^NS^ AMP: .3**	LI: −.1^NS^ AMP: .3*	1							
5. Number of problems last 6 months	LI: .4** AMP: .5***	LI: .3** AMP: .4***	LI: .4*** AMP: .5***	LI: .1^NS^ AMP: .1^NS^	1						
6. Organized support	LI: .3** AMP: .5***	LI: .2* AMP: .3**	LI: .3** AMP: .5***	LI: .2^NS^ AMP: .2^NS^	LI: .4*** AMP: .6***	1					
7. Community integration	LI: −.2* AMP: −.3**	LI: −.3* AMP: −.2*	LI: −.3* AMP: −.3*	LI: .0^NS^ AMP: −.1^NS^	LI: −.3* AMP: −.2*	LI: −.3* AMP: −.0^NS^	1				
8. Other‐perceived support	LI: .1^NS^ AMP: .3**	LI: .2^NS^ AMP: .2^NS^	LI: .1^NS^ AMP: .3*	LI: −.3** AMP: .0^NS^	LI: −.1^NS^ AMP: .3**	LI: −.1^NS^ AMP: .3**	LI: −.1^NS^ AMP: −.1^NS^	1			
9. Support received from nominee	LI: .1^NS^ AMP: .4***	LI: .1^NS^ AMP: .3**	LI: .1^NS^ AMP: .4***	LI: −.2* AMP: .1^NS^	LI: −.0^NS^ AMP: .4***	LI: −.0^NS^ AMP: .2^NS^	LI: −.0^NS^ AMP: −.1^NS^	LI: .6*** AMP: .4***	1		
10. Self‐efficacy	LI: −.4*** AMP: −.5***	LI: −.4** AMP: −.3*	LI: −.4*** AMP: −.4***	LI: .1^NS^ AMP: .1^NS^	LI: .1^NS^ AMP: −.3**	LI: −.1^NS^ AMP: −.3**	LI: .3* AMP: .2^NS^	LI: −.2* AMP: −.3***	LI: −.3* AMP: −.3**	1	
11. Language ability	LI: −.2* AMP: −.1^NS^	LI: −.1^NS^ AMP: −.0^NS^	LI: −.2^NS^ AMP: −.1^NS^	LI: .0^NS^ AMP: .0^NS^	LI: .1^NS^ AMP: .1^NS^	LI: .2^NS^ AMP: .0^NS^	LI: −.2^NS^ AMP: .2^NS^	LI: −.3** AMP: −.2*	LI: −.4*** AMP: −.1^NS^	LI: .2* AMP: .1^NS^	1
12. Non‐verbal ability	LI: −.2^NS^ AMP: −.2^NS^	LI: −.2^NS^ AMP: .0^NS^	LI: −.2^NS^ AMP: −.1^NS^	LI: .1^NS^ AMP: .2^NS^	LI: .2^NS^ AMP: .2^NS^	LI: .1^NS^ AMP: .0^NS^	LI: −.3* AMP: −.0^NS^	LI: −.4*** AMP: −.1^NS^	LI: −.4*** AMP: .1^NS^	LI: .4** AMP: .2*	LI: .5*** AMP: .3**

Note

AMP = age‐matched peer; LI = language impairment; NS = not significant.

*p < .05; **p < .01; ***p < .001.

For both groups, *the number of problems in the last 6 months* was correlated with depression and anxiety. This may be because experiencing more problems increases emotional health issues, or because mood disorders affect recall of problematic events and evoke more response to the PRQ85 items. Both groups also accessed more *organized support* the more depressed or anxious they felt. Higher depression and anxiety were also associated with *less integration into the community*. Again this factor is likely to be bidirectional. Higher *self‐efficacy scores* were associated with lower depression and anxiety scores for both groups.

For AMPs, there was a significant association between depression and *other‐perceived support*, but this was not the case for the LI group. Anxiety was not associated with other‐perceived support for either group. Neither were there any associations between depression and anxiety with *available support* (from PRQ85) and *the amount of regular help from the nominated person*.

Neither group showed correlations of note between concurrent language and emotional health scores. The only significant association was a small correlation (*r *=* *−.2) between language and depression for the young adults with LI. However, there were no differences in language ability between those who scored clinically on the BDI and those who did not.

### Statistical predictors of depression and anxiety

Two stepwise regression analyses were conducted: one with depression (BDI) and one with anxiety (BAI) as the dependent variable. Only variables which correlated significantly above were included as predictors. The final model for *depression* explained 38% of the variance, adj. *R* ^2^ = .38; *F*(4, 162) = 26.77, *p* < .001, with the predictors self‐efficacy, the number of problems experienced in the past 6 months, organized support, and group. Group was not significant in the final step. For *anxiety*, the final concurrent predictor model explained 27% of variance, adj. *R* ^2^ = .27; *F*(5, 160) = 13.30, *p* < .001, with the predictors self‐efficacy, the number of problems experienced in past 6 months, amount of available support, other‐perceived support and group, which again was not significant in the final step. Table 4 presents this information.

**Table 4:** Regression final model statistics: depression and anxiety

	*B*	*SE*	β	95% CI	*t*	*p*
Depression						
(Constant)	4.494	8.701			0.52	.606
Self‐efficacy	−0.608	0.106	−.376	−0.8, −0.4	−5.75	<.001
Number of problems in last 6 months	1.104	0.259	.301	0.6, 1.6	4.26	<.001
Organized support	1.593	0.592	.192	0.4, 2.8	2.69	.008
Group (LI/AMP)	−1.116	1.074	−.067	−3.2, 1.0	−1.104	.300
Anxiety						
(Constant)	15.337	3.996			3.09	.002
Self‐efficacy	−0.434	0.113	−.284	−0.7, −0.2	−3.86	<.001
Number of problems in last 6 months	1.128	0.235	.323	0.7, 1.6	4.79	<.001
Available support	0.147	0.048	.210	0.1, 0.3	3.08	.002
Other‐perceived support	0.612	0.311	.141	0.0, 1.2	1.97	.051
Group (LI/AMP)	−0.077	1.123	−.005	−2.3, 2.2	−0.07	.946

Note

AMP = age‐matched peer; LI = language impairment.

### Self‐efficacy as a mediator of emotional health differences

**Figure 1:**
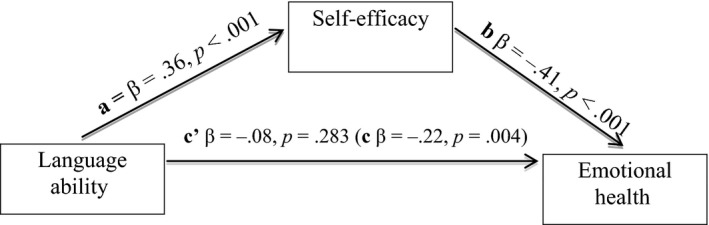
Self‐efficacy as a mediator between language and emotional health. a = positive relationship between language and self‐efficacy; b = negative relationship between self‐efficacy and emotional health; c = negative relationship between language and emotional health before considering self‐efficacy; c′ = absence of remaining relationship between language and emotional health once self‐efficacy has been added as a mediating factor.

Table 4 shows that self‐efficacy emerged as the primary predictor and was negatively associated with emotional health symptoms. Hence, it was investigated further using mediation analysis (Baron & Kenny, 1986). The mediation diagram is shown in Figure 1.

In this type of analysis, the effect of the mediator (self‐efficacy) on the dependent variable (emotional health; b) must be greater than the effect of the independent variable (language ability) on the DV (a); and the effect of the IV (language ability) on the DV (emotional health; c) should be significantly reduced or absent once the mediator (self‐efficacy) is controlled for (c′).

For the *overall sample*, there was a positive effect of language ability on self‐efficacy and negative effect of self‐efficacy on emotional health. There was no direct relationship between language ability and emotional health after this step. The zero‐order correlation between language ability and self‐efficacy was significant for the LI but not the AMP group. To test whether the mediation effect was different for the young adults with LI compared with the AMPs, the overall sample mediation model was re‐run and group was entered as a moderator in the relationship between language ability and self‐efficacy. Group was not a significant moderator (β = −.1, *p* = .448). Furthermore, the reverse pattern was not evident; that is, language was not a mediator for the effects of self‐efficacy on emotional health.

Therefore, for the overall sample, the relationship between language ability and emotional health is mediated by self‐efficacy. The mediation is not different between groups.

## Discussion

This study revealed four important findings: First, this sample of young adults with LI experienced higher levels of both depression and anxiety than their peers. Second, the amount of *available* support (including access to organized support such as third‐sector groups) was not different for adults with LI compared with AMPs. Third, social support was not significantly related to emotional health in those with LI; in contrast, for AMPs, uptake of support indicated poorer emotional health). Fourth, self‐efficacy mediated emotional health differences in both groups. These findings add to our knowledge of the likelihood of mental health difficulties in individuals with LI as they reach young adulthood and they enrich our understanding of key influential factors.

### Higher levels of mental health difficulties in young adults with LI

Higher levels of mental health difficulties were indicated at the symptom level. Higher than average symptom reporting is in line with some of the previous research which has shown higher levels depression, anxiety, and other psychiatric risk in adolescents and adults with LI using different measures (Clegg *et al*., 2005; Conti‐Ramsden & Botting, 2008). Higher levels of depression and anxiety symptoms have also been reported in other groups with developmental disorders, such as those with autism (Lugnegård *et al*., 2011) and ADHD (Nelson & Gregg, 2012) as they enter adulthood. Although our sample showed some evidence of increased prevalence of clinical‐level affective disorder (as indicated by scores over the clinical‐threshold), this finding has not been replicated in studies that have used diagnostic psychiatric interviews (Beitchman *et al*., 2014; Snowling *et al*., 2006). This inconsistency may indicate widespread subclinical difficulties, be caused by lower sensitivity of interview measures, or (as noted by Beitchman *et al*., 2014), reflect the nature of the individuals retained in long‐term longitudinal studies.

### Availability and receipt of social support

The amount of *available* support (including personal support as well as access to organized support such as third‐sector groups) was not different for adults with LI compared with AMPs. Furthermore, the nature of the support sought was not different across groups, with both samples relying on family and friends in the first instance. This is somewhat different to the pattern reported for younger people with LI regarding friendships and social activities (Durkin & Conti‐Ramsden, 2007) and for individuals with acquired aphasia (Hilari & Northcott, 2006; Northcott & Hilari, 2011) who report more dependence on family structures than on others of the same age. Nominated responders in our study reported that despite the similar levels of *available* support, adults with LI actually *received* more help from others, and this support was across more areas of functioning than for AMPs.

### Social support and emotional health

The relationship between social support and emotional health, however, was not straightforward. Whilst the groups reported experiencing the same number of problems in the last 6 months, different patterns of association with support were identified. For LI participants, emotional health was not significantly correlated with the amount of available support, the amount of help received from the nominee, or other‐perceived support. For AMPs, in contrast, higher levels of support were associated with *higher levels* of emotional health difficulties. Thus, it is difficult to unpick the protective role of support. Although a protective role has been seen in other studies of typical young individuals (Herman‐Stahl & Petersen, 1996), this finding has not always been replicated. Some researchers have found little association between support and emotional health problems (Dumont & Provost, 1999).

### Self‐efficacy

One of the most important findings of the present study was that self‐efficacy mediated emotional health differences across groups, with lower levels of depression and anxiety in individuals with higher self‐efficacy. Crucially, self‐efficacy was lower in adults with LI compared with peers. Self‐efficacy has been reported previously as an important factor in protecting against depression and anxiety in typical adolescents (Smith & Betz, 2002; Steca *et al*., 2014), adults (Rutter, 1985) and post‐stroke populations (van Mierlo, van Heugten, Post, de Kort, & Visser‐Meily, 2015). However, this is the first study to link self‐efficacy to emotional health in those with a history of developmental LI. The functional disadvantages of having poor language are likely to differ across different contexts (see Scott & Windsor, 2000 for a discussion of a continuum of difficulty by discourse genre). Nevertheless, it may be that self‐efficacy is lower when individuals live with the everyday challenges that are experienced by those with impoverished language. This is an important finding, because as young people with LI reach adulthood, specialist language and communication support from health and educational services is lacking. Furthermore, depressive symptoms in late adolescence and early adulthood have been shown to predict major depressive episodes in later life (Pine, Cohen, Gurley, Brook, & Ma, 1998). Understanding the protective role of self‐efficacy may mean, for example, that this should be targeted during the school years and late adolescence to help facilitate good emotional health in adulthood. In short, self‐efficacy bears on mental health in individuals with and without LI; but those with LI tend to have lower self‐efficacy, and thus are at greater risk of lacking the internal resources to manage their symptoms.

### Conclusion

This study used a large clinical cohort and comprehensive measurement to add to understanding of the factors concurrently predicting emotional health in a group of young adults with LI. In particular, it highlighted that developing self‐efficacy is likely to be a protective strategy and may be more important than providing additional social support *per se*. This is one of the few studies to investigate emotional health in young adults with developmental LI. However, the findings presented are not only relevant to clinical groups. Rather, they reveal a mediating role for self‐efficacy that is also significant for individuals without LI. The present research suggests that professionals and educators in contact with young people experiencing emotional health difficulties should investigate possible underlying language difficulties and facilitate counselling or other interventions aimed at developing robust self‐efficacy skills to protect against emotional disorder in those at risk.
